# The Predictive Role of Hand Section of Fugl–Meyer Assessment and Motor Activity Log in Action Research Arm Test in People With Stroke

**DOI:** 10.3389/fneur.2022.926130

**Published:** 2022-07-07

**Authors:** Peiming Chen, Tai-Wa Liu, Mimi M. Y. Tse, Claudia K. Y. Lai, Joshua Tsoh, Shamay S. M. Ng

**Affiliations:** ^1^Department of Rehabilitation Sciences, The Hong Kong Polytechnic University, Kowloon, Hong Kong SAR, China; ^2^School of Nursing & Health Studies, Hong Kong Metropolitan University, Ho Man Tin, Hong Kong SAR, China; ^3^School of Nursing, The Hong Kong Polytechnic University, Kowloon, Hong Kong SAR, China; ^4^School of Health Sciences, Yamaguchi University, Yamaguchi, Japan; ^5^Department of Psychiatry, Prince of Wales Hospital & Shatin Hospital, Shatin, Hong Kong SAR, China

**Keywords:** self-perceived performance, upper limb, motor function, stroke, assessment

## Abstract

**Background:**

Recent findings of clinical studies have demonstrated a significant positive relationship between Fugl–Meyer Assessment of upper extremity score and the action research arm test (ARAT) score in people with stroke. Although the motor activity log (MAL) can assess the self-perception of motor performance, which can affect the performance of the upper limb, the relationship between MAL score and ARAT score still remains unclear. The objective of this study is to quantify the independent contribution of MAL score and FMA-hand score on the ARAT score in people with stroke.

**Methods:**

This is a cross-sectional study. There were a total of 87 subjects (50 males, 37 females; mean age = 61.12 ± 6.88 years, post-stroke duration=6.31 ± 2.84 years) included in this study. Self-perceived performance in using the paretic limb was measured by MAL, including subscale of the amount of usage (MAL-AOU) and quality of movement (MAL-QOM). Functional performance of the upper limb was measured by action research arm test (ARAT). Upper limb motor control of the hand was measured by hand section of Fugl–Meyer assessment (FMA-hand).

**Results:**

The result showed that MAL-QOM (*r* = 0.648, *p* < 0.001), MAL-AOU (*r* = 0.606, *p* < 0.001), FMA-hand scores (*r* = 0.663, *p* < 0.001), and the use of a walking aid (*r* = −0.422, *p* < 0.001) were significantly correlated with the ARAT scores. A total 66.9% of the variance in the ARAT scores was predicted by the final regression model including MAL-QOM, MAL-AOU, FMA-hand scores, and walking aid. The FMA-hand score was the best predictor of ARAT scores, which can predict a 36.4% variance of ARAT scores in people with stroke, which controlled the effect of using a walking aid. After controlling for use of a walking aid and FMA-hand scores, the multiple linear regression modeling showed that MAL-QOM and MAL-AOU scores could also independently predict an additional 10.4% of the variance in ARAT scores.

**Conclusion:**

In addition to the FMA-hand score, the MAL score was significantly correlated with the ARAT score. Improving self-perceived performance should be one goal of rehabilitation in people with stroke. Further work developing and testing techniques to do so is clearly warranted.

## Introduction

Functional performance of the upper limb refers to the performance of using the arms and hands in the tasks of daily life (1). At least one-third of stroke survivors fail to regain full upper limb motor function within 6 months after their stroke (2–4) due to muscle weakness, abnormal muscle tone, or poor upper limb coordination (5). Improving the functional performance of the upper limbs and reducing the associated negative impacts in daily life are often the main goals of stroke rehabilitation (6, 7).

The action research arm test (ARAT) is a reliable and valid upper limb-specific instrument for evaluating the arm and hand functioning of people with neurological disorders, including stroke (8, 9), traumatic brain injury (10), multiple sclerosis (11, 12), and Parkinson's disease (13, 14). The ARAT quantifies the ability to grasp, grip, and pinch, and also perform gross arm movements with objects of different sizes, weights, and shapes.

Previous studies, Platz et al. (11), De Weerdt and Harrison (15), Rabadi and Rabadi (16), and Kwakkel and Kollen (17) have revealed that motor impairment limits the paretic upper limb's functioning in people with stroke. Muscle weakness, abnormal reflexes, and motor coordination, quantified using the Fugl–Meyer Assessment of the upper extremities (FMA-UE) score, were all significantly correlated with ARAT score in people with stroke (*r* = 0.77–0.925) (11, 15, 16). Furthermore, Kwakkel and Kollen (17) have shown that the hand subscale of the FMA (FMA-hand) is the best predictor of improvement in ARAT results in people with stroke (standardized β = 0.357; *p* ≤ 0.001). The arm, leg, and balance ability subscales show less predictive power (β ≤ 0.007; *p* ≤ 0.001). However, the independent contribution of FMA-hand to ARAT score has not been systematically investigated and quantified when the demographic data were also considered.

Self-perceived performance is a subjective feeling of how well and satisfied the people perceive their performance (18), rather than objective performance in real life. The low level of self-perceived performance is indeed associated with the objective performance in people with stroke (19, 20). Van der Lee et al. (19) has demonstrated a significant and moderate to good correlation (*r* = 0.63, *p* ≤ 0.001) between motor activity log (MAL) and ARAT scores in people with stroke. Poor self-perceived performance of the upper limb discourages the use of the paretic upper limb (21), which impedes the recovery of the limb's motor skills, leading to even less self-perceived performance, and a downward spiral in upper limb functioning, objectively measured. That makes it important to identify the individual contribution of self-perceived performance to real performance of upper limb in developing rehabilitation programs for people with stroke. However, no proper evaluation of that contribution has yet been published.

This study was therefore designed to determine (1) the correlation of ARAT score (functional performance of upper limb) with MAL (self-perceived performance) and FMA-hand (motor control of hand) scores in people with stroke; (2) whether the MAL score (self-perceived performance) and FMA-hand score (motor control of hand) can independently predict the ARAT score (functional performance of upper limb) in people with stroke, and if so, to quantify the individual contribution of MAL score (self-perceived performance) and FMA-hand score (motor control of hand) when sociodemographic factors are also considered.

## Materials and Methods

### Subjects

A total of 87 subjects were self-selected through poster advertising among local self-help groups. Those included (1) were between 50 and 80 years of age, (2) had been diagnosed with stroke at least 1 year previously, (3) had volitional control of the non-paretic arm, (4) could induce at least minimal anti-gravity movement in the shoulder of the paretic arm, (5) had at least 5° of wrist extension in the anti-gravity position, and (6) scored ≥7 (out of 10) on the Cantonese version of the Abbreviated Mental Test.

People were excluded if they (1) had any additional medical, cardiovascular, or orthopedic condition (e.g., angina pectoris), (2) had receptive dysphasia, (3) had visual impairment that could not be corrected by glasses (e.g., hemianopia), (4) had significant upper limb peripheral neuropathy, (5) had severe shoulder, elbow, wrist, or finger contractures that would preclude testing the arm's passive range of motion, or (6) were involved in other clinical trials.

### Study Design

This was a cross-sectional study. Ethical approval was obtained from the Ethics Committee of the Hong Kong Polytechnic University. The study was conducted in accordance with the Declaration of Helsinki (22).

### Procedures

The assessments were performed in the neurorehabilitation laboratory of the Hong Kong Polytechnic University. After obtaining the subjects' written informed consent, they completed a socio-demographic questionnaire and then were assessed with all the tests administered in random order within the same day. All of the tests were administered by a physiotherapist with 5 years of clinical experience. All of the instruments used had previously been validated in the local context (23, 24).

### Outcome Measure

#### Action Research Arm Test (ARAT)

The ARAT (25) was used to assess the functional performance of each subject's paretic upper limb. The ARAT scores function on an ordinal scale with 19 items, each rated from 0 to 3 with “0 = no movement,” “1 = movement task is partially performed,” “2 = movement task is completed but takes abnormally long,” or “3 = movement task is performed normally.” The total score thus ranges from 0 to 57. According to the guidelines (26), the subjects are asked to perform the most difficult task within a subscale first. If they complete it successfully and get a score of 3 on that task, then all the other items within that subscale are also scored as 3. A score between 0 to 2 on the first item indicates that the second item (easiest) should be evaluated. If the subject scores 0 on the second item, then the rest of the items within that subscale are also scored as 0. Otherwise, the rest of the tasks within the subscale are administered. A previous study, Van der Lee et al. (27) has shown that the ARAT has excellent intra-rater (*r* = 0.996–0.997) and inter-rater (*r* = 0.989) reliability in assessing people with chronic stroke.

#### Motor Activity Log (MAL)

The quality of movement (QOM) and amount of usage (AOU) subscales of MAL were used to quantify self-perceived performance in using a paretic upper limb. Each consist of 30 items quantifying the subject's self-perceived performance in using a paretic upper limb in life situations during the previous week, such as turning on a light and brushing the teeth (28). Each of the 30 items is rated as 0 (never), 1 (very poor), 2 (poor), 3 (fair), 4 (almost normal), or 5 (normal) in QOM. For AOU, the ratings are 0 (not used), 1 (very rarely), 2 (rarely), 3 (half of the pre-stroke frequency), 4 (3/4 of the pre-stroke frequency), or 5 (the same as before the stroke). Higher scores indicate higher self-perceived performance in using the paretic upper limb. Both the MAL-QOM and MAL-AOU have demonstrated good test–retest reliability [QOM: intraclass correlation coefficient (ICC) = 0.82; AOU: ICC = 0.79] (29) and excellent internal consistency (QOM: Chronbach's α = 0.87; AOU: Chronbach's α > 0.82) (30) in assessing people with stroke.

#### Fugl–Meyer Assessment-Hand Function (FMA-Hand)

Motor control of the paretic hand was assessed using the FMA-hand instrument. It consists of 7 items (items 24–30 of the FMA-UE) with a total score of 14. It assesses motor control of finger flexion and extension, thumb adduction, finger opposition, cylindrical grip, and spherical grip using ratings of 0, 1, or 2. Higher scores indicate better motor control of the paretic hand. The entire FMA-UE has shown excellent intra-rater (ICC = 0.984–0.993) and inter-rater (ICC = 0.995–0.996) reliability when used to assess people with stroke (31).

### Statistical Analysis

The data were analyzed using version 22.0 of the Statistical Package for the Social Sciences software. Descriptive statistics were compiled summarizing the demographic information and the FMA-UE, FMA-hand, MAL-QOM, MAL-AOU, and ARAT scores. Kolmogorov–Smirnov test was used to evaluate the normality of the data distributions. We first investigated the correlations between ARAT score and all variables to identify the variables which showed a significant correlation with ARAT score in people with stroke. Pearson or Spearman correlation coefficients were then computed to evaluate the strength of the relationships between ARAT scores with FMA-hand, MAL-AOU, MAL-QOM scores, and the demographic data, as appropriate. To control for socio-demographic differences, partial correlation coefficients were used to estimate the association between ARAT score with the FMA-hand, MAL-QOM, and MAL-AOU scores. The variables which demonstrated a significant correlation with the ARAT score were further analyzed with multiple linear regression. Their individual power in predicting the ARAT score was determined by a multiple linear regression model with the forced entry method. The significance level was set at 0.05 (two-tailed).

## Results

### Subject Characteristics

Thirty-seven females (43%) and fifty males (57%) with a mean age of 61.12 ± 6.88 years and a mean BMI of 24.07 ± 3.79 kg/m^2^ were recruited. Their mean post-stroke duration was 6.31 ± 2.84 years. Among them, 47 (54%) had left hemiplegia and 40 (46%) had right hemiplegia. Forty-nine of the subjects had suffered an infarction while the other 38 had survived hemorrhagic strokes. Seven of the subjects lived alone while the others lived with family. Sixty-eight of the subjects used a walking aid and the others could walk without an aid. The group's mean FMA-UE score was 34.51 ± 11.69. The mean FMA-hand score was 7.55 ± 2.84. The mean MAL-QOM score and MAL-AOU score were 39.35 ± 37.77 and 29.61 ± 30.84, respectively (Table 1).

**Table 1:** Demographic information describing the subjects.

**Baseline information**	**Mean ±SD**
Age (years)	61.12 ± 6.88
BMI (kg/m^2^)	24.07 ± 3.79
Post-stroke duration (years)	6.31 ± 2.84
ARAT score	23.76 ± 16.62
FMA-UE score	34.51 ± 11.69
FMA-hand score	7.55 ± 2.84
MAL-QOM score	39.35 ± 37.77
MAL-AOU score	29.61 ± 30.84
	**Number of subjects**
Gender (female/male)	37/50
Paretic side (left/right)	47/40
Type of stroke (infarct/hemorrhage)	49/38
Living situation (live alone/live with family)	7/80
Walking aid (yes/no)	68/19

*N = 87.*

*SD, standard deviation; BMI, body mass index; ARAT, Action Research Arm Test; FMA-UE, Fugl–Meyer Assessment for the Upper Extremities; FMA-hand, hand subscale of Fugl–Meyer Assessment; MAL-QOM, Motor Activity Log Quality of Movement subscale; MAL-AOU, Motor Activity Log Amount of Usage subscale*.

### Relationships Between ARAT Scores and the Other Outcome Measures

**Figure 1:**
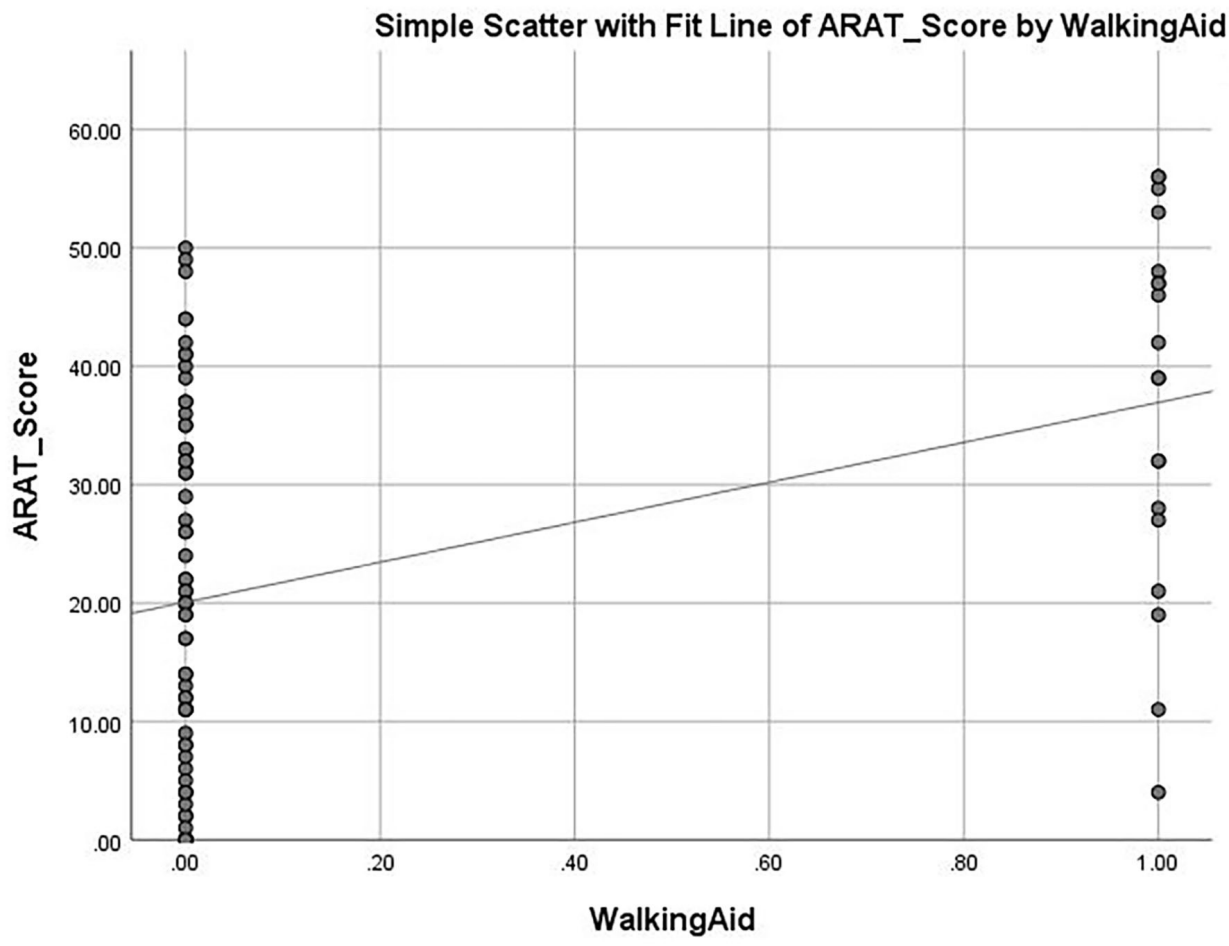
The correlation between ARAT score and the use of walking aid.

**Figure 2:**
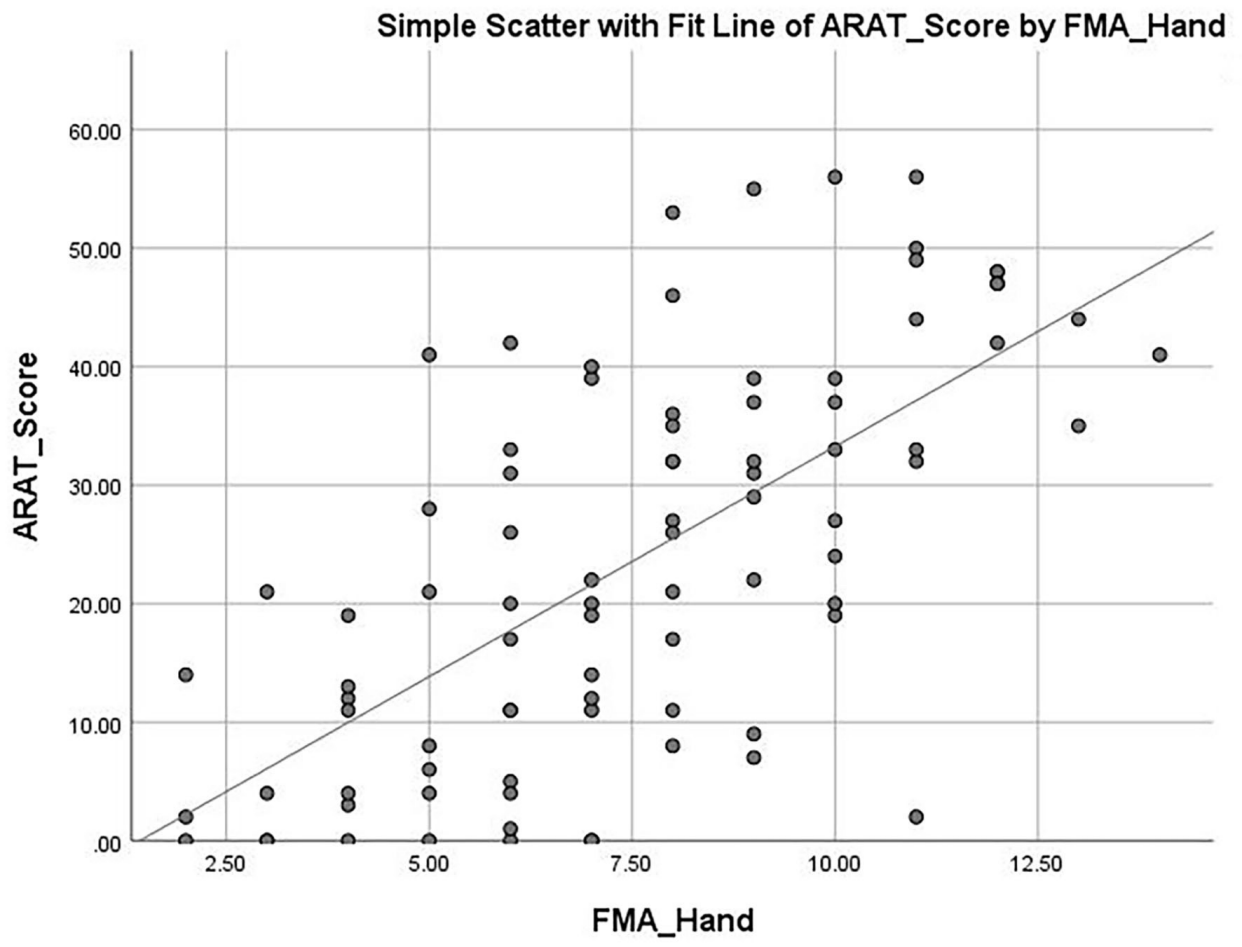
The correlation between ARAT score and hand section of Fugl-Meyer Assessment score.

**Figure 3:**
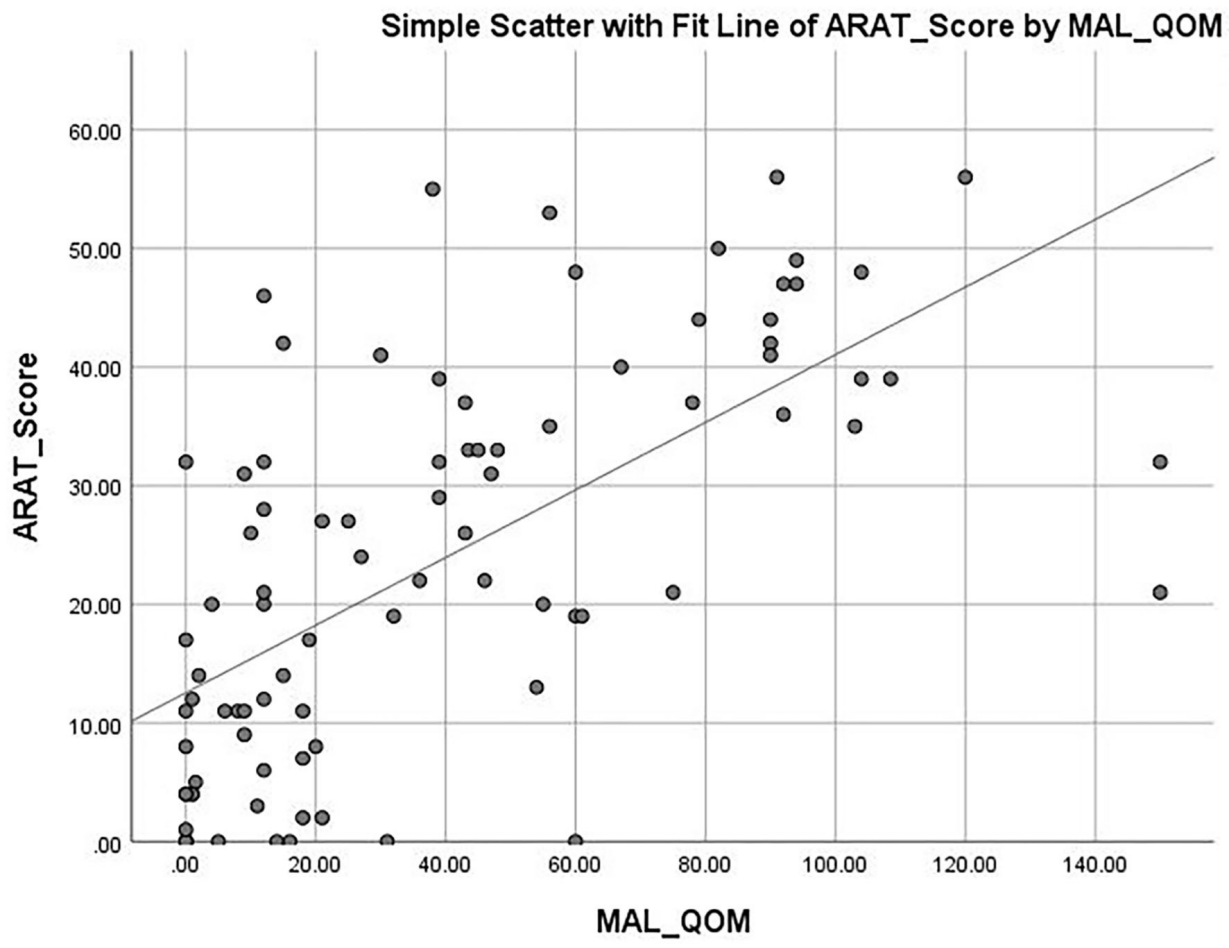
The correlation between ARAT score and MAL-QOM score.

**Figure 4:**
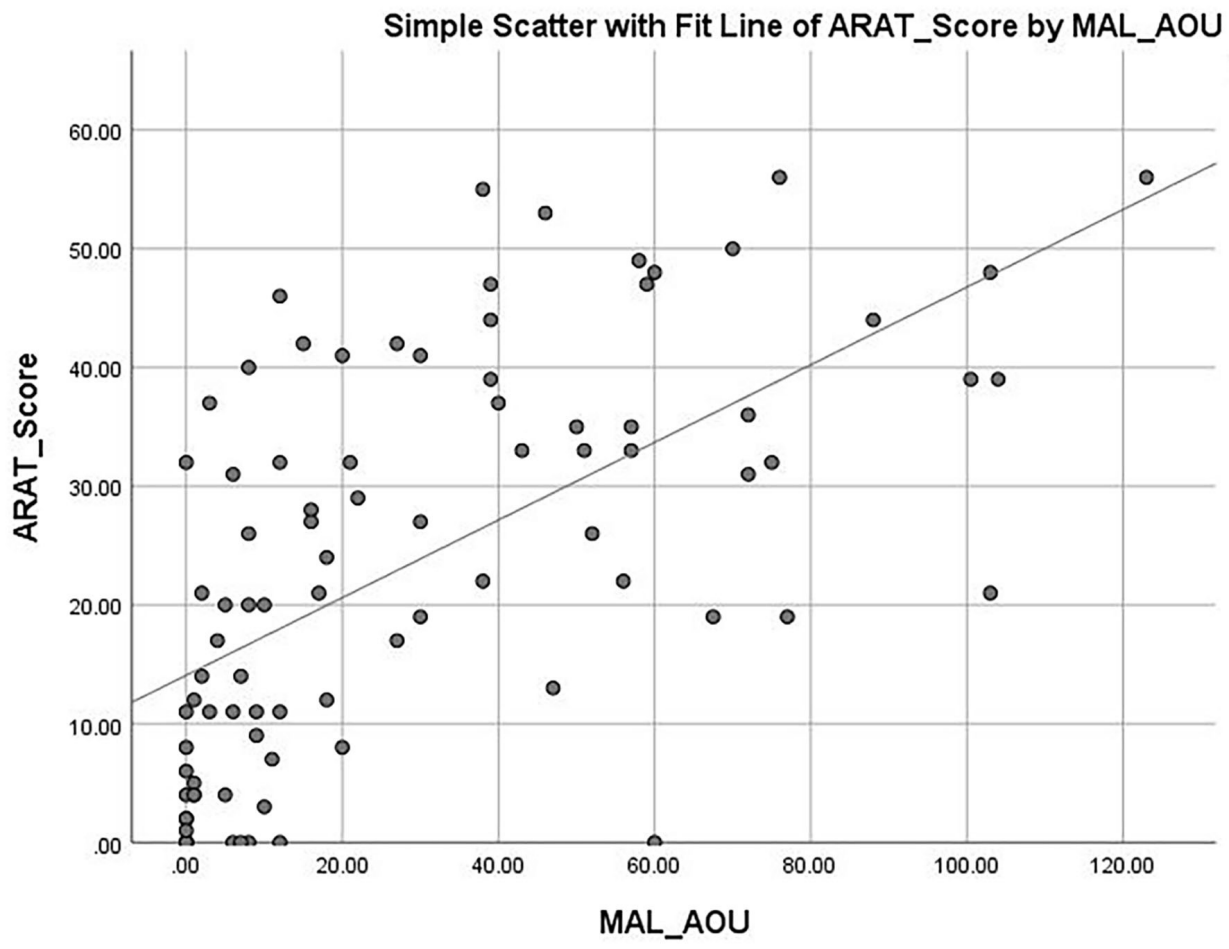
The correlation between ARAT score and MAL-AOU score.

The mean ARAT score of 23.76 ± 16.62 indicates that the subjects had a “moderate” level of functional performance of the upper limb, on average. A published Rasch analysis has concluded that an ARAT score between 22 and 42 should be defined as moderate level of functional performance (32). The ARAT score were significantly correlated with the use of a walking aid (*r* = 0.422, *p* ≤ 0.001; Figure 1), the FMA-hand (*r* = 0.663, *p* ≤ 0.001; Figure 2), MAL-QOM (*r* = 0.648, *p* ≤ 0.001; Figure 3), and MAL-AOU scores (*r* = 0.606, *p* ≤ 0.001; Figure 4; Table 2). After controlling for the use of a walking aid, strong and significant partial correlation coefficients were found between the ARAT score and the FMA-hand (*r* = 0.680, *p* ≤ 0.001), MAL-QOM (*r* = 0.606, *p* ≤ 0.001), and MAL-AOU scores (*r* = 0.551, *p* ≤ 0.001; Table 3).

**Table 2:** The correlation between ARAT score and the other variables.

**Demographic information**	**Correlation coefficients (r)**
Age	0.121
Gender	−0.121
BMI	−0.189
Post-stroke duration	0.076
Paretic side	0.048
Type of stroke	−0.059
Living situation	0.048
Walking aid use	−0.422**
FMA-hand score	0.663**
MAL-QOM score	0.648**
MAL-AOU score	0.606**

*N = 87.*

*ARAT, Action Research Arm Test; BMI, body mass index; FMA-hand, hand subscale of Fugl–Meyer Assessment; MAL-QOM, Motor Activity Log Quality of Movement subscale; MAL-AOU, Motor Activity Log Amount of Usage subscale.*

^**^Indicates a correlation significant at the p ≤ 0.001 level of confidence, r indicated Pearson correlation.

**Table 3:** Partial correlation coefficients (controlling for using a walking aid) between ARAT scores and other variables.

**Outcome measure**	**Partial correlation coefficient**
FMA-hand score	0.680**
MAL-QOM score	0.606**
MAL-AOU score	0.551**

*N = 87.*

*FMA-hand, hand subscale of Fugl–Meyer Assessment; MAL-QOM, Motor Activity Log Quality of Movement subscale; MAL-AOU, Motor Activity Log Amount of Usage subscale.*

^**^Indicates a correlation significant at the p ≤ 0.001 level of confidence.

### Contributions of FMA-Hand, MAL-QOM, and MAL-AOU Scores to ARAT Score

After conducting the partial correlation, we found that use of walking aid significantly correlated with the ARAT score. To control the effect of use of walking aid in the regression model, we put the use of walking alone in model 1. To show the independent predictive power of FMA-hand score to ARAT score, we put the FMA-hand scores and the use of walking aid in model 2. To show the independent predictive power of the self-perceived performance, we put MAL-QOM, MAL-AOU, FMA-hand score, and use of walking aid in model 3.

Table 4 shows the predictive power of the different variables for ARAT score as determined by multiple linear regression analysis with the forced entry method. The full model (*F*_3, 83_ = 44.490, *p* ≤ 0.001) was able to explain 66.9% of the variance in the ARAT score. The FMA-hand score (β = 0.610) was the best predictor of ARAT score (model 2, Table 4) with the highest Pearson correlation coefficient (*r* = 0.663, *p* ≤ 0.001). After controlling for use of a walking aid and FMA-hand results, the multiple linear regression modeling (model 3) showed that MAL-QOM (β = 0.238) combined with MAL-AOU (β = 0.174) could independently predict an additional 10.4% of the variance in ARAT score. The Pearson correlation coefficients were MAL-QOM: *r* = 0.648, *p* ≤ 0.001; MAL-AOU: *r* = 0.606, *p* ≤ 0.001.

**Table 4:** Relationships between ARAT scores and other variables.

	**Adjusted *R*^2^**	***R*^2^ change**	**Predictor**	***B***	**95% confidence interval**	**β**	***p*-Value**
Model 1	0.201	0.201	Use of a walking aid	−18.355	−26.018 to −10.693	−0.459	≤ 0.001
Model 2	0.565	0.364	Use of a walking aid	−14.864	−20.577 to −9.151	−0.372	≤ 0.001
			FMA-hand score	3.571	2.735 to 4.407	0.610	≤ 0.001
Model 3	0.669	0.104	Use of a walking aid	−10.919	−16.124 to −5.715	−0.273	≤ 0.001
			FMA-hand score	2.645	1.835 to 3.455	0.452	≤ 0.001
			MAL-QOM score	0.105	0.020 to 0.189	0.238	0.016
			MAL-AOU score	0.094	−0.007 to 0.195	0.174	0.069

*B, unstandardized regression coefficient; β, standardized regression coefficient; FMA-hand, hand subscale of Fugl–Meyer Assessment; MAL-QOM, Motor Activity Log-quality of movement subscale; MAL-AOU, Motor Activity Log Amount of Usage subscale*.

## Discussion

### Summary

To the best of our knowledge, this has been the first published study to investigate the individual contribution of MAL (self-perceived performance) and FMA-hand scores (motor control of hand) on the ARAT score (functional performance of upper limb) in people with stroke, revealing that stroke survivors with a lower perception of function (MAL) showed poorer motor function (ARAT). That finding adds to the current knowledge about the roles of MAL and FMA-hand score in ARAT score in stroke rehabilitation. The clinical implication is that improving self-perceived performance in using paretic upper limb could enhance actual upper limb functional performance among people with stroke.

### ARAT and MAL Scores

A previous study, Hoonhorst et al. (32) used ARAT score to classify performance as no capacity (ARAT score: 0–10), poor capacity (11–21), limited capacity (22–42), notable capacity (43–54), or full capacity (ARAT score 55–57). In this study, the mean ARAT score was 23.76, and the ARAT score of most subjects (35.6%) fell into the pool between 22 and 42, which indicates limited functional performance of the paretic upper limb in people with stroke.

The mean MAL-QOM and MAL-AOU scores were 39.35 ± 37.77 and 29.61 ± 30.84, which means that the average score on each item was 1.31 and 0.99, respectively. According to the guidelines (29), those averages indicate a relatively low level of self-perceived performance. Two other studies (19, 33) reported similar findings in people with stroke. The low performance in MAL score would be expected to influence a person's willingness to use the paretic upper limb (34). Using it less will tend to worsen its actual performance as measured by FMA-hand (35), feeding back to MAL scores in a potential downward spiral.

### MAL Score Predicts Performance of ARAT Score

The full model predicted 66.9% of the variance in the ARAT score. FMA-hand score and MAL score were significant and independent predictors of the ARAT score, accounting for 36.4 and 10.4% of the variance, respectively. These findings are consistent with those of previous studies showing that FMA-hand score are associated with the ARAT score among people with stroke (17, 36). This study is the first to demonstrate that MAL-QOM and MAL-AOU scores are independent predictors of ARAT score in people with stroke.

In Bandura's theory (37), decisions about activity and behavior could be influenced by one's beliefs about the ability to engage in them successfully. Bandura and Adams suggested that the influence is partly cognitive, and people predict specific behavioral consequences and their attitudes based on those perceptions. In this study, those having a better perception of their performance in using their paretic upper limb were more likely to use it in their daily lives. The practice of the paretic upper limb would help them maintain or even improve their proficiency. More practice of the paretic limb in the real-life would help the people with stroke to maintain or even improve their proficiency in daily activity skills with the paretic upper limb. Conversely, the people with stroke who have low self-perceived performance in using their paretic limb would probably avoid using it to some extent. It resulted in less motor control in the long term (38). That could explain why the MAL score were significant predictors of ARAT score in people with stroke.

A total of 33.1% of the variance in the ARAT score remained unexplained in the full model. Several psychological and physical factors which were not included could explain that. Some psychological factors like fatigue (39, 40) and depression (41) were not accounted for in this study's design. In addition, physical factors, such as upper limb muscle weakness (42), spasticity (43), limited range of motion (44), impaired sensation (45), and hand dominance prior to the stroke (33) were also not included in this study. Future studies investigating the contributions of all these psychological and physical factors on ARAT score are certainly warranted.

### Correlations of Other Parameters With ARAT Score

The analyses showed that using a walking aid was a significant predictor of ARAT score, while age, gender, BMI, post-stroke duration, paretic side, type of stroke, and living situation showed no significant predictive power. The explanation could be that using a walking aid indicates poor motor control of the upper limb reflected in a poor ARAT score.

In this study, the type of stroke did not show significant correlation with ARAT score. It could be explained by the subjects' post-stroke stages, which should influence the progress of neural recovery. Andersen et al. (46) has reported that people with hemorrhagic stroke are more likely to have a poorer prognosis in the acute phase than those who have survived an ischemic one because the lesioned area is generally more extensive. However, as spontaneous recovery after hemorrhage and ischemia progresses, people may regain comparable levels of upper limb function. That would tend to explain the lack of any significant association between ARAT scores and types of stroke in this study.

### Clinical Implications

In this study, a significant correlation has been identified between the ARAT score with the MAL and FMA-hand scores. These findings may indicate that the upper limb rehabilitation program targeted to enhance self-perceived performance could lead to the improvement of the upper limb function as measured by ARAT score in people with stroke. The goal should, of course, be to encourage greater and more frequent use. For example, physiotherapists could incorporate “graded” activity training into the customary physical training. The grades could boost commitment to using the paretic side by giving positive feedback. More frequent, more active use should eventually improve self-perceived performance (47). Further, longitudinal study with larger sample size verifying the proposed causal relationship is warranted.

### Limitations

The final model of multiple linear regression (model 3, Table 4) accounted for 66.9% of the total variance in the ARAT score, leaving 33.1% of the variance unexplained. Future studies should investigate other factors such as depression and mental fatigue. This cross-sectional study was conducted with a small sample size, and stronger relationships could be inferred in further studies with a larger sample size having different degrees of upper limb impairments. In addition, the subjects were all self-selected Chinese volunteers recruited from local self-help groups. That always raises the possibility that they were untypically active and relatively less impaired than typical stroke survivors. The study's strict inclusion and exclusion criteria also limits the generalizability of the results. Hand dominance may also play an important role in the perception of the motor function (33), which was not investigated in our study. Complemental studies are warranted to investigate the effect of hand dominance on self-perceived performance.

## Conclusions

Our results demonstrate that the FMA-hand score can usefully predict ARAT score in people with chronic stroke. The MAL-QOM and MAL-AOU scores are significant independent predictors of the ARAT score. Thus, improving self-perceived performance could be one of the rehabilitation goals in people with stroke. Further work developing and testing the intervention protocol to improve self-perceived performance in stroke survivors is warranted.

## Data Availability Statement

The raw data supporting the conclusions of this article will be made available by the authors, without undue reservation.

## Ethics Statement

The studies involving human participants were reviewed and approved by the Hong Kong Polytechnic University. The patients/participants provided their written informed consent to participate in this study.

## Author Contributions

Study design: SN, PC, and CL. Data collection and data analysis: PC. Manuscript writing and result interpretation: PC, T-WL, MT, CL, JT, and SN. All authors contributed to the article and approved the submitted version.

## Funding

This study was supported by the Health and Medical Research Fund (17182001), Food and Health Bureau, Hong Kong SAR Government, awarded to SN and her team.

## Conflict of Interest

The authors declare that the research was conducted in the absence of any commercial or financial relationships that could be construed as a potential conflict of interest.

## Publisher's Note

All claims expressed in this article are solely those of the authors and do not necessarily represent those of their affiliated organizations, or those of the publisher, the editors and the reviewers. Any product that may be evaluated in this article, or claim that may be made by its manufacturer, is not guaranteed or endorsed by the publisher.

## Abbreviations

AOU: amount of use
ARAT: action research arm test
FMA-UE: Fugl–Meyer assessment of upper extremity
FMA-hand: hand section of Fugl–Meyer Assessment
MAL: motor activity log
ICC: intraclass correlation coefficient
QOM: quality of movement
BMI: body mass index.
